# Lic regulates JNK‐mediated cell death in *Drosophila*

**DOI:** 10.1111/cpr.12593

**Published:** 2019-03-07

**Authors:** Yihao Sun, Di Zhang, Chenglin Li, Jiuhong Huang, Wenzhe Li, Yu Qiu, Aiwu Mao, Mingcheng Zhou, Lei Xue

**Affiliations:** ^1^ The First Rehabilitation Hospital of Shanghai, Shanghai Key Laboratory of Signaling and Diseases Research, School of Life Science and Technology Tongji University Shanghai China; ^2^ International Academy of Targeted Therapeutics and Innovation Chongqing University of Arts and Sciences Chongqing China; ^3^ Tongren Hospital Shanghai Jiao Tong University School of Medicine Shanghai China

**Keywords:** cell death, Egr, JNK, Lic, MKK3

## Abstract

**Objectives:**

The evolutionary conserved JNK pathway plays crucial role in cell death, yet factors that modulate this signalling have not been fully disclosed. In this study, we aim to identify additional factors that regulate JNK signalling in cell death, and characterize the underlying mechanisms.

**Materials and Methods:**

*Drosophila* were raised on standard media, and cross was carried out at 25°C. The Gal4/UAS system was used to express proteins or RNAi in a specific temporal and spatial pattern. Gene expression was revealed by GFP fluorescence, X‐gal staining or immunostaining of 3rd instar larval eye and wing discs. Cell death was visualized by acridine orange (AO) staining. Images of fly eyes and wings were taken by OLYMPUS microscopes.

**Results:**

We found that *licorne* (*lic*) encoding the *Drosophila* MKK3 is an essential regulator of JNK‐mediated cell death. Firstly, loss of *lic* suppressed ectopic Egr‐triggered JNK activation and cell death in eye and wing development. Secondary, *lic* is necessary for loss‐of‐cell polarity‐induced, physiological JNK‐dependent cell death in wing development. Thirdly, Lic overexpression is sufficient to initiate JNK‐mediated cell death in developing eyes and wings. Furthermore, ectopic Lic activates JNK signalling by promoting JNK phosphorylation. Finally, genetic epistatic analysis confirmed that Lic acts in parallel with Hep in the Egr‐JNK pathway.

**Conclusions:**

This study not only identified Lic as a novel component of the JNK signalling, but also disclosed the crucial roles and mechanism of Lic in cell death.

## 1 INTRODUCTION

Programmed cell death (PCD), or apoptosis, is a fundamental biological phenomenon in which cell death is genetically and biochemically regulated. Dysregulation of apoptosis will trigger a series of disorders that result in many types of diseases such as neurodegenerative diseases, immunodeficiency diseases and tumours.^1^, ^2^, ^3^, ^4^ Apoptosis is a strictly controlled process involving a series of gene activation, while expression and regulation of these genes are highly conserved among species, such as Bcl‐2 family, Caspase family and oncogene such as *c‐myc* 5 and tumour suppressor gene *p53*.^6^ The JNK signalling is a very important pathway involved in the regulation of cell death,^7^ migration, proliferation^8^ and differentiation,^7^ as well as cell morphology maintenance, cytoskeleton construction and other biological processes.^1^, ^9^ *Drosophila *is one of the best model organisms to study genetics, and many of the JNK pathway components and regulators have been identified from genetic screens in *Drosophila*.^10^, ^11^, ^12^

The stress‐activated protein kinase (SAPK) signalling consists of the JNK and p38 pathways. Its primary function is to respond to a series of environmental stresses (nutrients, osmotic pressure, temperature, etc) and participate in the regulation of apoptosis, proliferation, differentiation and other responses to adapt to changes in the external environment to ensure the proper function of the body.^13^ In *Drosophila*, p38 is activated via dual phosphorylation at the Thr‐Gly‐Tyr motif by a specific MAPKK, Licorne (Lic), which encodes the fly ortholog of MKK3,^14^ whereas the fly JNK, Bsk, is phosphorylated and activated by *Hemipterous *(*Hep*) encoding the MKK7 ortholog. In 2009, Caroline Baril et al^15^ found that mutant of Alphabet (Alph), a serine/threonine phosphatase belongs to the *Drosophila* protein phosphorylation 2C (PP2C) family, was able to rescue the lethality caused by *hep* or *lic* mutation. Genetic epistatic analysis confirmed that Alph acts as a negative regulator upstream of Hep and Lic in the MAPK pathway. In addition, both p38 and JNK signalling could be triggered by similar activators, such as pro‐inflammatory cytokines (TNFα and IL‐1) and stress stimulation (UV, H_2_O_2_ and heat shock).^16^ These results suggest that the two pathways may have overlapping or redundant functions, which might be achieved by sharing component(s). However, despite the reported role of Lic in p38 signalling, it remains elusive whether Lic also regulates JNK signalling in vivo.

We have previously carried out a genetic screen in *Drosophila* to search for additional regulators of Egr‐triggered JNK‐mediated cell death.^12^, ^17^ In this study, we provide genetic evidences demonstrating that *lic* encodes an essential component of the Egr‐JNK pathway involved in cell death. We found that Lic is necessary for ectopic Egr‐induced or loss‐of‐cell polarity‐induced JNK activation and cell death. Ectopic Lic is sufficient to promote JNK phosphorylation, which activates JNK signalling and triggers cell death in development. Genetics data suggest Lic acts in parallel with Hep as a JNK kinase. However, it deserves further investigation whether MKK3 could phosphorylate and activate JNK signalling in mammal.

## 2 MATERIALS AND METHODS

### 2.1 *Drosophila* stocks and genetics

Stocks were raised on standard *Drosophila* media, and crosses were performed at 25°C. The following stocks were described previously: *w^1118^* 18; *TRE‐RFP* 19; *GMR*‐Gal4, *ey*‐Gal4, *pnr*‐Gal4, *ptc*‐Gal4, *sd*‐Gal4*, UAS*‐GFP, *UAS*‐Hep^20^; *hh*‐Gal4^21^; *sev*‐Gal4, *UAS*‐dTAK1^22^; *UAS*‐Wnd^23^; *UAS‐GFP‐RNAi*, *UAS*‐Puc, *UAS*‐Bsk^DN^ 24; *UAS*‐Lic, *UAS*‐Lic^KD^ 25; *UAS*‐Egr^26^; *puc^E69^* 27; and *UAS*‐*scrib‐RNAi*, *UAS*‐p35.^11^ *hs*‐Gal4 (#1799), *UAS*‐LacZ (#3956), *UAS*‐*p38b‐RNAi* (#29405), *UAS‐lic‐RNAi *(#31643), *UAS*‐*p38a‐RNAi* (#27316), *UAS*‐*p38c‐RNAi* (#64846), *UAS‐mkk4‐RNAi *(#42832) and *UAS*‐Bsk (#9310) were obtained from the Bloomington Drosophila Stock Center. *UAS‐lic‐RNAi *(#20166), *UAS‐wnd‐RNAi *(#13786)^28^ and *UAS‐hep‐RNAi* (#26929)^27^ were received from the Vienna Drosophila RNAi Center. *UAS‐dTAK1‐RNAi *(#1388R‐2) and *UAS‐p38b‐RNAi *(#7393R‐1) were acquired from the National Institute of Genetics (NIG), Japan. *tub*‐Gal80, *hs‐Flp*, *FRT19A*; *act*‐Gal, *UAS*‐GFP (FRT19A tester) was a gift from Prof. Lei Zhang. *FRT19A, lic^d13^* mutant fly stock was a gift from Prof. Haiyun Song and has been previously described.^29^ *lic* mutant clones were generated with the MARCM system 30 and labelled by GFP expression (*lic^d13^*, *FRT19A*/*tub*‐Gal80, *hs‐Flp*, *FRT19A*; *act*‐Gal4, *UAS*‐GFP/+). Flp recombinase was expressed conditionally using *hs‐Flp*. Heat shocks were performed at 37°C for 15 minutes during the first or second instar larval stage.

### 2.2 Antibodies

The following primary antibodies were used for immunostaining: rabbit anti‐phospho‐JNK (1:200; Calbiochem, San Diego, CA) and mouse anti‐β‐Gal (1:500; DSHB). The second antibodies were used as follows: anti‐mouse CY3 (1:1000; Cell Signaling Technology, Danvers, MA, USA) and anti‐rabbit CY3 (1:1000; CST). The following primary antibodies were used for Western blot analysis: rabbit anti‐p‐JNK (9251S, 1:1000; CST) and rabbit anti‐JNK (sc‐7345, 1:500; Santa Cruz Biotechnology, Dallas, TX, USA).

### 2.3 X‐gal staining

Eye and wing discs were dissected from third instar larvae in PBST (1× PBS pH 7.0, 0.1% Triton X‐100) and stained for β‐galactosidase activity as described.^31^

### 2.4 AO staining

Eye and wing discs were dissected from 3rd instar larvae in 1× phosphate‐buffered saline (PBS) and incubated in 1 × 10^−5^ mol/L acridine orange (AO) for 5 minutes at room temperature.^17^

### 2.5 Image of fly eyes and wings

Three‐day‐old flies were collected and frozen at −80°C. When taking pictures, flies were unfrozen at room temperature and placed on 1% agarose plate. Light images of eye were taken by OLYMPUS stereo microscope SZX16 (Olympus Corporation, Shinjuku, Tokyo, Japan). Wings were dissected and placed on slide with alcohol/glycerol (1:1) buffer. Light images of wing were taken by OLYMPUS BX51 microscope.

## 3 RESULTS

### 3.1 Lic is essential for ectopic Egr‐induced cell death in eye development

**Figure 1:**
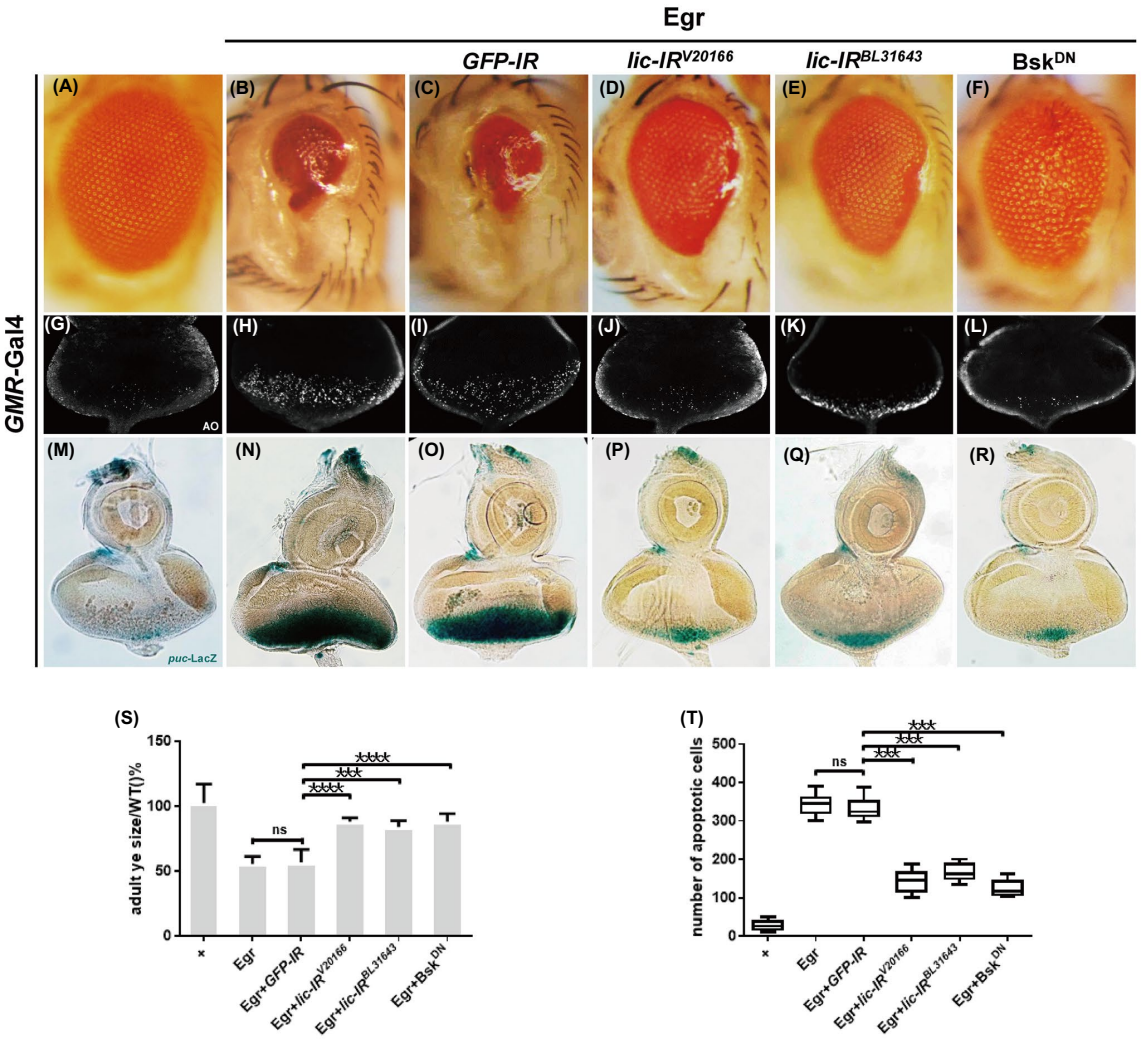
Depletion of *lic *suppressed Egr‐induced cell death and JNK activation in eye development. Light micrographs of *Drosophila* adult eyes (A‐F) are shown. Compared with *GMR*‐Gal4 control eyes (A), overexpression of Egr activated cell death and produced a distinct small eye phenotype (B), which was not affected by the expression of a *GFP‐RNAi* (C), but was partially inhibited by the expression of two independent *lic‐RNAi* (D, E), and strongly blocked by the expression of Bsk^DN^ (F). Fluorescent micrographs of third instar eye discs (G‐L) are shown. Compared with the control eye disc (G), *GMR*>Egr triggered extensive cell death posterior to the MF (H), which was not inhibited by expressing a *GFP‐RNAi* (I), but was blocked by expressing two *lic‐RNAi* (J, K) or Bsk^DN^(L). Light micrographs of third instar eye discs with X‐gal staining (M‐R) are shown. Compared with the *GMR*‐Gal4 control (M), overexpression of Egr activated *puc*‐LacZ expression posterior to the MF (N), which was suppressed by expressing two *lic‐RNAi* (P, Q) or Bsk^DN^ (R), but not a *GFP‐RNAi* (O). (S) Quantification of adult eye sizes, which have been normalized to the *GMR*‐Gal4 control, was shown, *n* ≥ 15. (T) Statistic analysis of AO‐positive cell numbers, *n* ≥ 12. One‐way ANOVA test was used to calculate statistical significance, mean + SD, ns, *P* > 0.05; *P < 0.05; **P < 0.01; ***P < 0.001; ****P < 0.0001. Genotypes: (A, G) *GMR*‐Gal4/+ (B, H) *UAS*‐Egr/+; *GMR*‐Gal4/+ (C, I) *UAS*‐Egr/*UAS*‐*GFP‐IR*; *GMR*‐Gal4/+ (D, J) *UAS*‐Egr/*UAS‐lic‐IR^V20166^*; *GMR*‐Gal4/+ (E, K) *UAS*‐Egr/+; *GMR*‐Gal4/*UAS‐lic‐IR^BL31643^* (F, L) *UAS*‐Egr/+; *GMR*‐Gal4/*UAS*‐Bsk^DN ^(M) *GMR*‐Gal4/+; *puc*‐LacZ/+ (N) *GMR*‐Gal4, *UAS*‐Egr/+; *puc*‐LacZ/+ (O) *GMR*‐Gal4, *UAS*‐Egr/*UAS‐GFP‐IR*; *puc*‐LacZ/+ (P) *GMR*‐Gal4, *UAS*‐Egr/*UAS*‐*lic‐IR^V20166^*; *puc*‐LacZ/+ (Q) *GMR*‐Gal4, *UAS*‐Egr/+; *puc*‐LacZ/*UAS*‐*lic‐IR^BL31643^* (R) *GMR*‐Gal4, *UAS*‐Egr/+; *puc*‐LacZ/*UAS*‐Bsk^DN^

Egr is the fly ortholog of tumour necrosis factor (TNF)^32^ and a well‐recognized upstream regulator of the JNK signalling pathway.^33^ Egr is reported to play important roles in the regulation of cell proliferation, differentiation, apoptosis and immunity.^34^ Compared with the control (Figure 1A,G), ectopic expression of Egr in the developing eye driven by *GMR*‐Gal4 (*GMR*>Egr) triggers JNK‐mediated cell death in the eye imaginal disc (Figure 1H,T) and produces a small eye phenotype in the adult (Figure 1B,S).^35^ We found that Egr‐triggered small eye phenotype was effectively suppressed by knocking‐down *lic *with two independent RNAi lines (Figure 1D,E,S), but remained unaffected by expressing a *GFP‐RNAi *control transgene (Figure 1C,S).

Expression of the *lic‐RNAi* by itself did not produce any discernible eye phenotype (Figure S1a,b). *lic^d13^* is a P element‐induced allele of *lic*, with a deletion of 1411 nucleotides that removes the initiating methionine and the first 117 amino acids of the *lic *coding sequence.^29^ Consistent with the RNAi data, *GMR*>Egr‐induced small eye phenotype was effectively suppressed in heterozygous *lic^d13^* mutants (Figure S2b,e). As a positive control, blocking JNK signalling by expressing a dominant negative form of Bsk (Bsk^DN^), the fly ortholog of JNK, significantly inhibited Egr‐induced eye defect (Figure 1F,S). Consistent with the adult eye phenotypes, Egr‐induced cell death in the eye disc, indicated by acridine orange (AO) staining, was markedly inhibited by mutation or RNAi‐mediated depletion of *lic* (Figure 1J,K,T and Figure S2d,f) and Bsk^DN ^(Figure 1L), but not that of *GFP‐RNAi *(Figure 1I). Together, these data suggest that Lic is necessary for ectopic Egr‐induced cell death in *Drosophila* eye development.

### 3.2 Lic is required for ectopic Egr‐induced JNK pathway activation in developing eyes

Given that Lic is required for Egr‐induced JNK‐dependent cell death, we wonder whether Lic is necessary for Egr‐induced JNK pathway activation. To this end, we examined the expression of *puc*‐LacZ, a well‐known reporter that characterizes the activity of JNK signalling.^35^, ^36^ Compared with control discs (Figure 1M), *GMR*>Egr induced strong *puc*‐LacZ expression posterior to the morphogenetic furrow (MF) in eye discs (Figure 1N), which was significantly suppressed by expressing two *lic‐RNAi *(Figure 1P,Q) or Bsk^DN ^(Figure 1R), but not the *GFP‐RNAi* (Figure 1O). Thus, Lic is necessary for ectopic Egr‐induced JNK signalling activation in developing eyes.

### 3.3 Lic is necessary for Egr‐induced JNK‐mediated cell death in wing development

**Figure 2:**
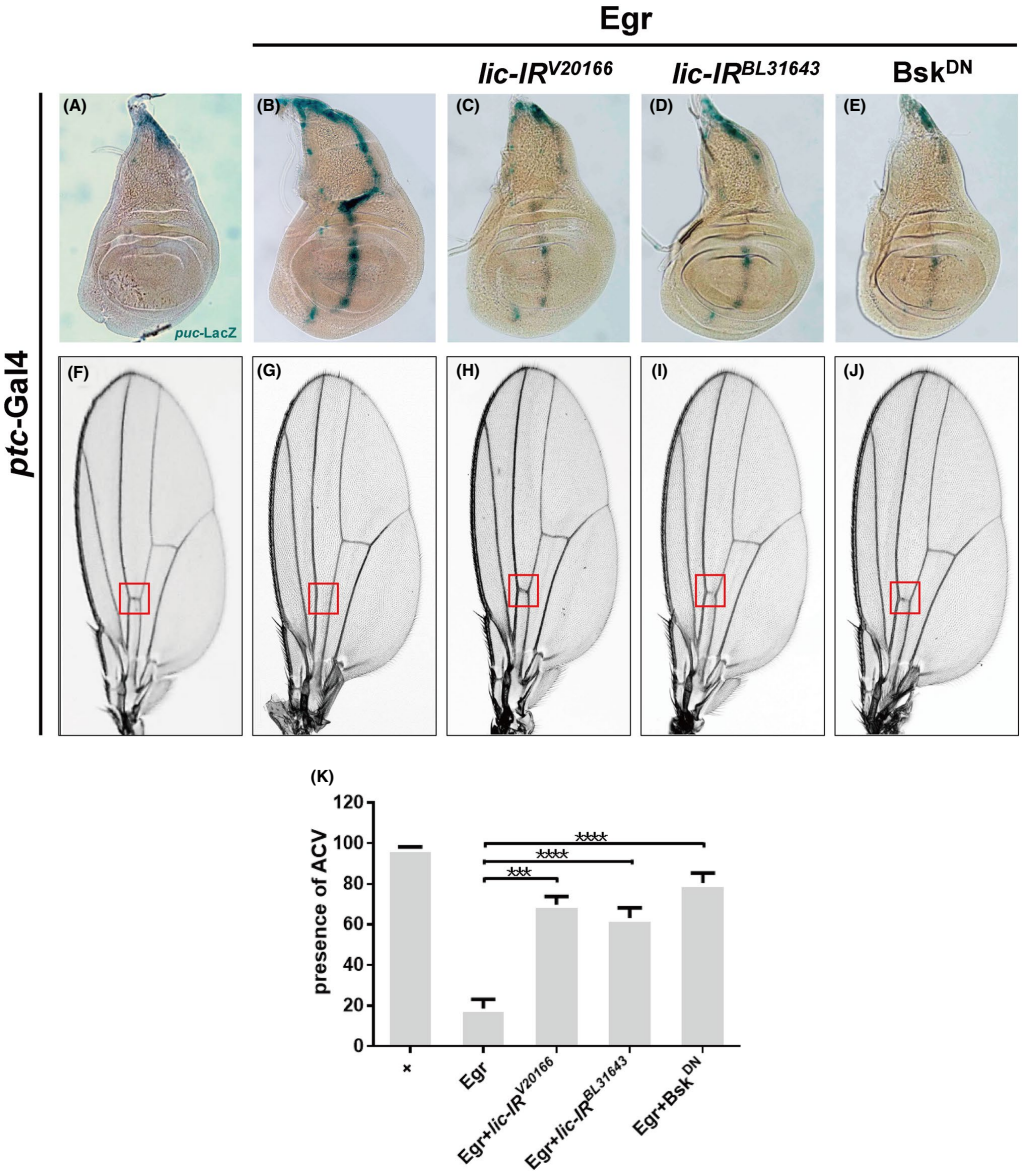
*Lic *is required for ectopic Egr‐induced JNK activation and cell death in wing development. Light micrographs of third instar wing discs (A‐E) and adult wings (F‐J) are shown. Compared with the *ptc*‐Gal4 controls (A, F), ectopic expression of Egr along the A/P boundary triggered *puc*‐LacZ expression in the wing disc (B) and produced a loss‐of‐ACV phenotype in the adult wing (G, the ACV area is indicated by a red box), both of which were suppressed by expressing two *lic‐RNAi *(C, D, H, I) or Bsk^DN^ (E, J). (K) The presence of ACV was quantified and shown. One‐way ANOVA test was used to calculate statistical significance, n > 20, mean + SD, ***P < 0.001; ****P < 0.0001. Genotypes: (A) *ptc*‐Gal4/+; *puc*‐LacZ/+ (B) *ptc*‐Gal4, *UAS*‐Egr/+; *puc*‐LacZ/+ (C) *ptc*‐Gal4, *UAS*‐Egr/*UAS*‐*lic‐IR^V20166^*; *puc*‐LacZ/+ (D) *ptc*‐Gal4, *UAS*‐Egr/+; *puc*‐LacZ/*UAS*‐*lic‐IR^BL31643^* (E) *ptc*‐Gal4, *UAS*‐Egr/+; *puc*‐LacZ/*UAS*‐Bsk^DN^ (F) *ptc‐*Gal4*/+* (G) *ptc*‐Gal4, *UAS*‐Egr/+ (H) *ptc*‐Gal4, *UAS*‐Egr/*UAS*‐*lic‐IR^V20166^* (I) *ptc*‐Gal4, *UAS*‐Egr/+;* UAS*‐*lic‐IR^BL31643^*/+ (J) *ptc*‐Gal4, *UAS*‐Egr/+; *UAS*‐Bsk^DN^/+

To investigate whether Lic modulates Egr‐induced JNK activation and cell death in other tissues, we turned to the wing imaginal disc—another in vivo system that has been widely used to study signal transduction pathway. Ectopic expression of Egr driven by *ptc*‐Gal4 (*ptc*>Egr) along the anterior/posterior compartment boundary (A/P boundary) in wing discs triggered JNK activation (Figure 2B) and cell death,^22^ which resulted in the loss of anterior cross vein (ACV) in adult wings (Figure 2G), as compared with controls (Figure 2A,F). We found that knock‐down *lic* significantly suppressed Egr‐induced *puc*‐LacZ expression (Figure 2C,D) and the loss‐of‐ACV phenotype (Figure 2H,I) to a similar extent as expression of Bsk^DN^ (Figure 2E,J,K), while expression of *lic‐RNAi* by itself did not produce any obvious wing phenotype (Figure S3a,b). These results suggest that Lic modulates Egr‐induced JNK‐dependent cell death in a non–tissue‐specific manner.

### 3.4 Lic is required for physiological JNK‐mediated cell death

**Figure 3:**
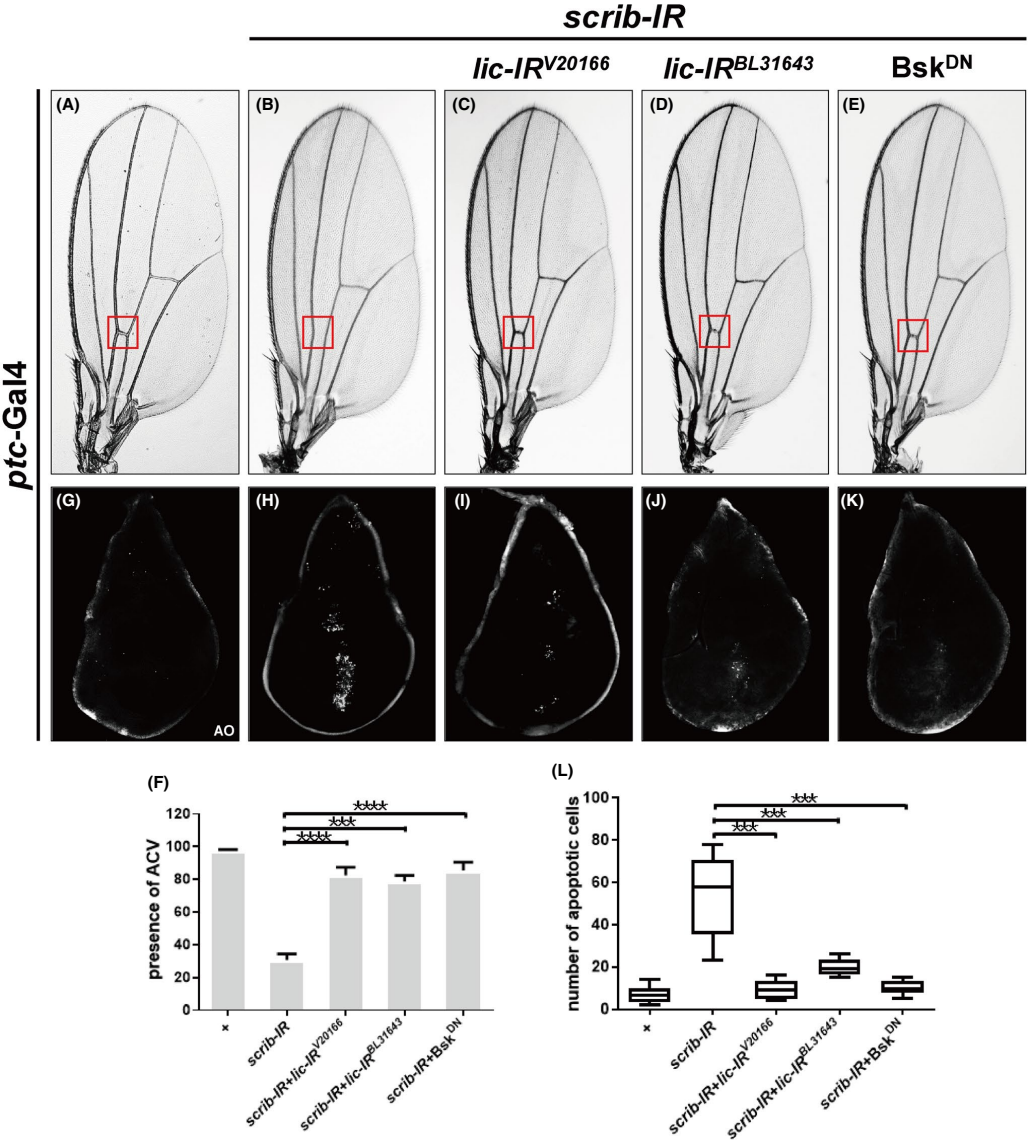
*Lic* is necessary for physiological JNK‐induced cell death. Light micrographs of adult wings (A‐E) and fluorescent micrographs of third instar wing discs (G‐K) are shown. Compared with the *ptc*‐Gal4 controls (A, G), depletion of *scrib *induced a loss‐of‐ACV phenotype in adult wings (B) and robust cell death in wing discs (H), both of which were suppressed by expressing two *lic‐RNAi* (C, D, I, J) or Bsk^DN^ (E, K). The ACV area is indicated by a red box. Statistical analysis for the presence of ACV (f, n > 30) or AO‐positive cell numbers (L, *n* ≥ 10) was shown. One‐way ANOVA test was used to calculate statistical significance, mean + SD, ***P < 0.001; ****P < 0.0001. Genotypes: (A, G) *ptc‐*Gal4*/+* (B, H) *ptc*‐Gal4, *UAS‐scrib*‐IR/+ (C, I) *ptc*‐Gal4, *UAS‐scrib*‐IR/*UAS*‐*lic‐IR^V20166^* (D, J) *ptc*‐Gal4, *UAS‐scrib*‐IR/+;* UAS*‐*lic‐IR^BL31643^*/+ (E, K) *ptc*‐Gal4, *UAS‐scrib*‐IR/+; *UAS*‐Bsk^DN^/+

The above data suggest that Lic is necessary for ectopic Egr‐induced JNK activation and cell death. Next, we wanted to verify whether Lic is involved in physiological activation of JNK signalling. Previous studies have found that disruption of cell polarity caused JNK‐mediated cell death.^37^, ^38^ Under the control of the *ptc *promoter, RNAi‐mediated downregulation of *scrib* generated the loss‐of‐ACV phenotype in adults (Figure 3B,F), which is resulted from extensive cell death at the A/P boundary of the wing disc (Figure 3H,L). We found that depletion‐of‐*scrib‐*induced ACV loss and cell death were effectively inhibited by knocking‐down *lic* or expressing Bsk^DN^ that served as a positive control (Figure 3C‐F,I‐L). Collectively, these data suggest that Lic is essential for physiological JNK‐mediated cell death.

### 3.5 Lic sufficiently activates JNK signalling

To investigate whether Lic is sufficient to activate JNK signalling, we overexpressed Lic in various regions of the wing disc. Ectopic expression of Lic along the A/P boundary driven by *dpp*‐Gal4 (Figure S4a) or in the wing pouch by *sd*‐Gal4 (Figure S4d) significantly upregulated *puc*‐LacZ expression in the corresponding regions (Figure S4c,f), as compared with GFP‐expressing controls that show endogenous *puc*‐LacZ expression in the dorsal tip (Figure S4b,e). A binding site for AP‐1 transcription factor in tetradecanoylphorbol acetate (TPA) has been referred to as TPA response element (TRE). A *TRE*‐RFP transgene has been served as another reporter to reveal JNK activity in vivo.^19^ Compared with the GFP‐expressing control, ectopic Lic expression driven by *hh*‐Gal4 in the posterior compartment of wing discs dramatically enhanced *TRE*‐RFP expression (Figure S4g‐i). Taken together, overexpression of Lic is sufficient to activate JNK signalling in vivo.

### 3.6 Lic regulates JNK phosphorylation

**Figure 4:**
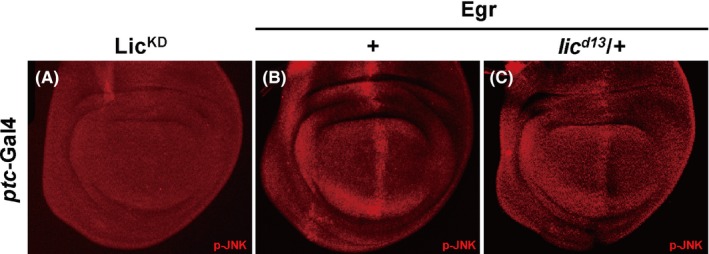
Heterozygous *lic* mutation suppresses ectopic Egr‐induced JNK phosphorylation in the wing disc. Fluorescent micrographs of *Drosophila* third instar wing discs (A‐C) are shown. Expression of a kinase‐dead form of Lic (Lic^KD^) fails to trigger JNK phosphorylation (A). Egr expression induces strong JNK phosphorylation (B), which is partially suppressed in *lic* heterozygous mutants (C). Genotypes: (A) *ptc*‐Gal4/+; *UAS*‐Lic^KD^/+ (B) *ptc*‐Gal4/*UAS*‐Egr (C) *lic^d13^*/+; *ptc*‐Gal4/*UAS*‐Egr

**Figure 5:**
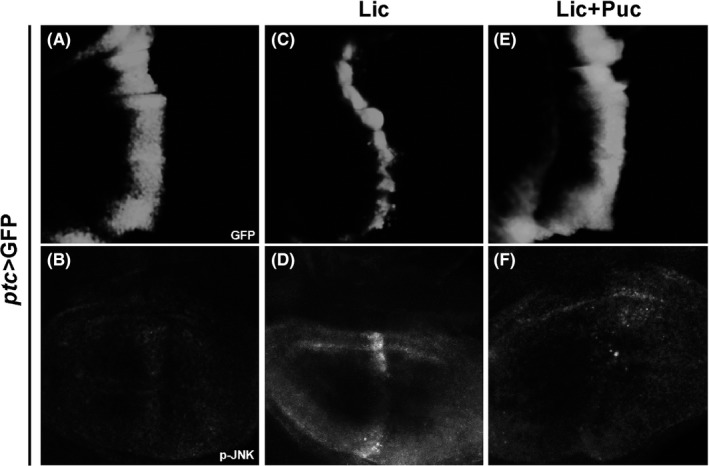
Lic promotes JNK phosphorylation. Fluorescent micrographs of wing discs with p‐JNK antibody staining are shown. Compared with the control (A, B), Lic overexpression promoted JNK phosphorylation (C, D), which was impeded by Puc expression (E, F). Genotypes: (A, B) *ptc*‐Gal4, *UAS*‐GFP/+ (C, D) *ptc*‐Gal4, *UAS*‐GFP/+; *UAS*‐Lic/+ (E, F) *ptc*‐Gal4, *UAS*‐GFP/+; *UAS*‐Lic/*UAS*‐Puc

In *Drosophila*, JNK is known to be activated through phosphorylation by the MKK7 ortholog Hep.^39^ While Lic encodes the *Drosophila* ortholog of MKK3 that has been implicated in the p38 signalling,^14^, ^40^ its role in JNK signalling has not been previously reported. We found that *ptc*>Egr‐induced JNK phosphorylation (Figure 4B) was significantly suppressed by mutation of *lic* (Figure 4C), suggesting Lic is required for Egr‐induced JNK phosphorylation. To investigate whether Lic could trigger JNK phosphorylation, we overexpressed Lic along the A/P boundary in the wing disc and checked JNK phosphorylation by a p‐JNK antibody. Compared with the GFP‐expressing control (Figure 5A,B), ectopic Lic was able to trigger JNK phosphorylation along the A/P boundary in the wing pouch (Figure 5C,D), which was further confirmed by Western blot analysis (Figure S5). Intriguingly, the width of the GFP stripe was significantly reduced upon Lic expression, presumably due to Lic‐induced cell death that was blocked by co‐expression of p35 (Figure S6d). Puc is a serine/threonine protein phosphatase that blocks JNK activity through dephosphorylation. We found that expression of Puc dramatically suppressed ectopic Lic‐induced JNK phosphorylation and restored the GFP stripe width (Figure 5E,F). Collectively, these data suggest that Lic regulates Egr‐induced JNK phosphorylation.

### 3.7 Lic induces JNK‐dependent cell death in development

Given that Lic can sufficiently activate JNK signalling, we wonder whether Lic is able to elicit JNK‐dependent cell death. To this end, we expressed Lic along the A/P boundary in 3rd instar wing discs by *ptc*‐Gal4. Previous work reported that activation of JNK by *ptc*>Hep resulted in cell death in wing discs and ACV loss in adult wings.^41^

**Figure 6:**
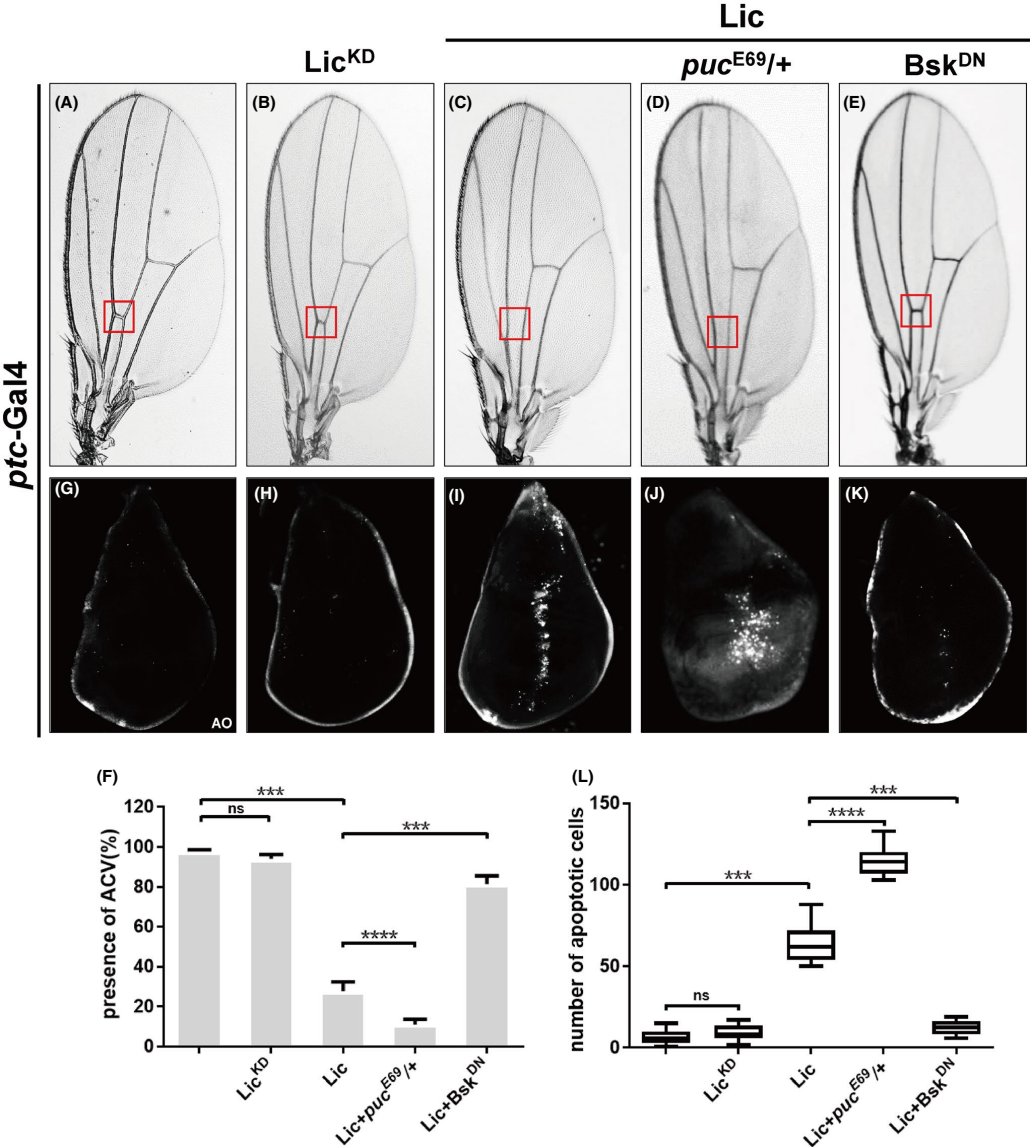
Lic induces JNK‐dependent cell death in wing development. Light micrographs of adult wings (A‐E) and fluorescent micrographs of third instar wing discs (G‐K) are shown. Compared with controls (A, G), overexpression of Lic driven by *ptc*‐Gal4 caused loss of ACV in adults (C) and strong cell death in wing discs (i). Both phenotypes were enhanced in heterozygous *puc* mutants (D, J) and impeded by expressing Bsk^DN^ (E, K). Expression of a kinase‐dead version of Lic (Lic^KD^) failed to trigger cell death and loss‐of‐ACV (B, H). The ACV area is indicated by a red box. The presence of ACV in adult wings (F) and number of apoptotic cells in wing discs (L) was quantified, *n* ≥ 16. One‐way ANOVA test was used to calculate statistical significance, mean + SD, ns, *P* > 0.05; ***P < 0.001; ****P < 0.0001. Genotypes: (A, G) *ptc*‐Gal4/+ (B, H) *ptc*‐Gal4/+; *UAS*‐Lic^KD^/+ (C, I) *ptc*‐Gal4/+; *UAS*‐Lic/+ (D, J) *ptc*‐Gal4/+; *puc^E69^*/*UAS*‐Lic (E, K) *ptc*‐Gal4/+; *UAS*‐Bsk^DN^/*UAS*‐Lic

We found that, compared with the *ptc*>GFP control (Figure 6A,G), *ptc*>Lic was able to trigger cell death in wing discs and produce the loss‐of‐ACV phenotype in adults (Figure 6C,I). Both phenotypes could be further enhanced by a mutation in the endogenous *puc*, but blocked by the expression of Bsk^DN^ (Figure 6D‐F,J‐L). Intriguingly, expression of MKK3 was able to trigger JNK‐dependent cell death in the wing disc (Figure S7). Collectively, the data suggest that Lic is sufficient to induce JNK‐dependent cell death in wing development, and this function is retained by MKK3.

To test whether Lic promotes JNK‐mediated cell death in other tissues, we ectopically expressed Lic in the developing eye or scutellum by the *ey*‐Gal4 or *pnr*‐Gal4 driver. Compared with the controls, expression of Lic resulted in reduced organ sizes, which were significantly suppressed by expressing Bsk^DN^ or Puc (Figure S8a,c‐f and g, i‐l), indicating that Lic triggers JNK‐dependent cell death in a non–tissue‐specific manner. Importantly, expressing a kinase‐dead version of Lic (Lic^KD^) 42 failed to trigger JNK phosphorylation (Figure 4A) and cell death (Figure 6H) or produce any discernible phenotypes (Figure 6B and Figure S8b,h), suggesting the kinase activity is indispensable for Lic to induce JNK‐mediated cell death.

### 3.8 Lic acts in parallel with Hep to promote JNK‐mediated cell death

**Figure 7:**
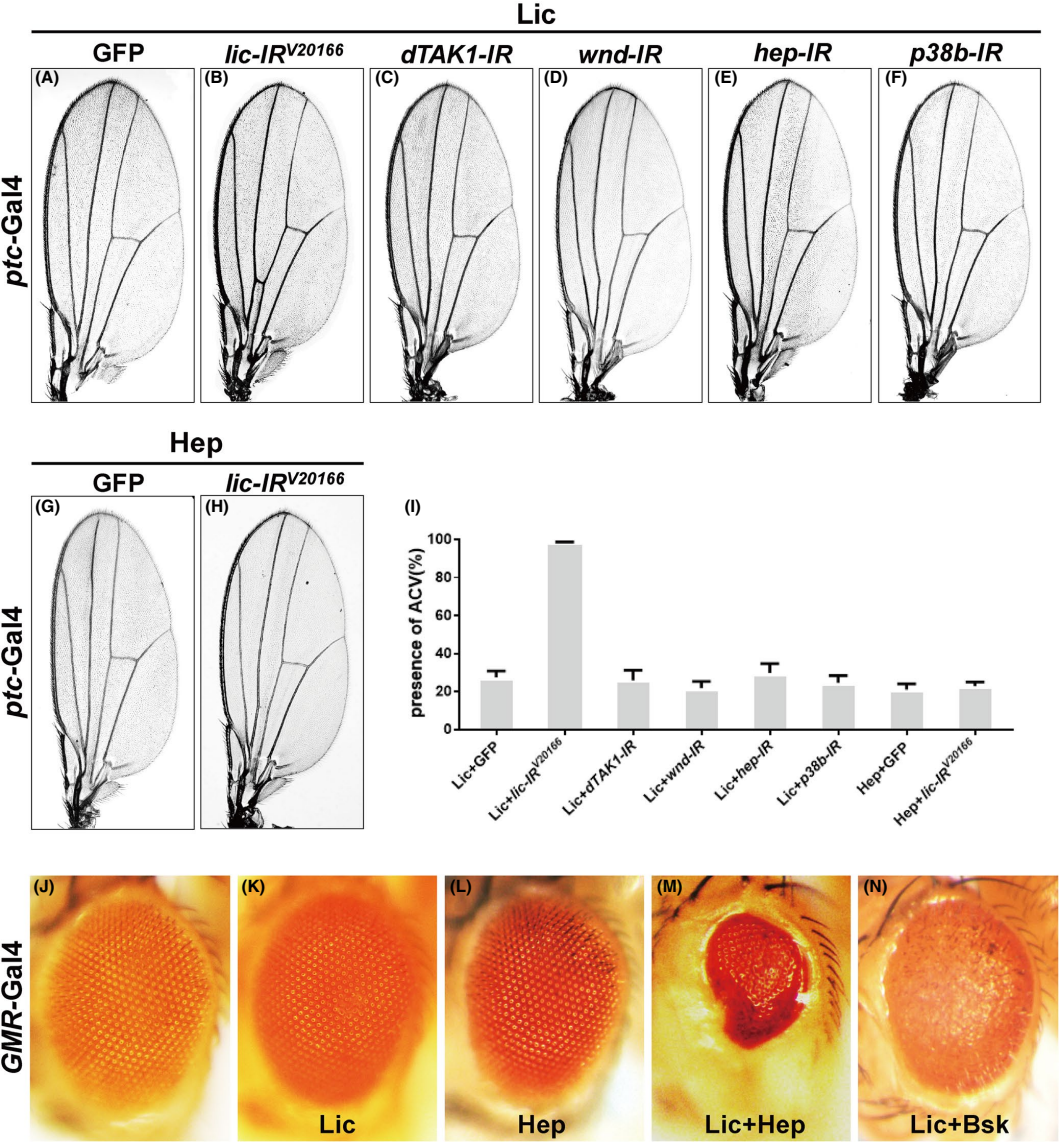
Lic acts in parallel to Hep to promote JNK‐mediated cell death. Light micrographs of *Drosophila* adult wings (A‐H) and eyes (J‐M) are shown. Ectopic Lic‐triggered ACV loss was blocked by expressing a *lic‐*RNAi (B), but remained unaffected by expressing GFP (A), or RNAi of *dTAK1* (C), *wnd* (D), *hep* (E) or *p38b *(F). Ectopic expression of Hep produced a similar loss‐of‐ACV phenotype, which was not suppressed by expressing GFP (G) or *lic *RNAi (H). The presence of ACV in adult wings was quantified (I). Compared with the *GMR*‐Gal4 control (J), expression of Lic (K) or Hep (L) alone in the eye produced no obvious phenotype, while co‐expression of Lic and Hep resulted in reduced eye size (M). Co‐expression of Lic and Bsk did not affect the eye size (N). Genotypes: (A) *ptc*‐Gal4/+; *UAS*‐Lic/*UAS*‐GFP (B) *ptc*‐Gal4/*UAS*‐*lic‐IR^V20166^*; *UAS*‐Lic/+ (C) *ptc*‐Gal4/*UAS*‐*dTAK1‐IR*; *UAS*‐Lic/+ (D) *ptc*‐Gal4/+; *UAS*‐Lic/*UAS‐wnd‐IR* (E) *ptc*‐Gal4/+; *UAS*‐Lic/*UAS*‐*hep‐IR* (F) *ptc*‐Gal4/+; *UAS*‐Lic/*UAS*‐*p38b‐IR* (G) *ptc*‐Gal4, *UAS*‐Hep/+; +/*UAS*‐GFP (H) *ptc*‐Gal4, *UAS*‐Hep/*UAS*‐*lic‐IR^V20166^* (J) *GMR*‐Gal4/+ (K) *GMR*‐Gal4/+; *UAS*‐Lic/+ (L) *GMR*‐Gal4/Hep (M) *GMR*‐Gal4/Hep; *UAS*‐Lic/+ (N) *GMR*‐Gal4/+; *UAS*‐Lic/*UAS*‐Bsk

So far, our data suggest Lic is an essential component that acts downstream of Egr but upstream of Bsk in the Egr‐JNK pathway. As both Lic (*Drosophila *MKK3) and Hep (*Drosophila* MKK7) belong to the mitogen‐activated protein kinase family (MAPKKs), and both are able to promote JNK phosphorylation, we assume Lic may act in parallel with Hep, and downstream of the MAPKKKs dTAK1 and Wnd.^23^ To test this hypothesis, we performed genetic epistasis analysis between Lic and known kinases in the Egr‐JNK pathway by using the loss‐of‐ACV phenotype, which had been the most constant and sensitive phenotype in our hands. As mentioned before, the ACV loss phenotype produced by *ptc*>Lic was significantly inhibited by expressing Bsk^DN^ (Figure 6C,E), indicating Lic acts upstream of Bsk. In addition, this phenotype was fully suppressed by expressing two independent *lic‐RNAi*, but not GFP (Figure 7A,B,I and Figure S9b), which served as the positive and negative controls, respectively. Consistent with the hypothesis, Lic‐induced loss‐of‐ACV phenotype was not suppressed by depleting *dTAK1*, *wnd, hep* or *mkk4*, and *vice versa*, Hep‐induced ACV loss could not be recovered by depleting *lic *(Figure 7C‐E,G‐I and Figure S9c). Furthermore, ectopic expression of Lic or Hep driven by *GMR*‐Gal4 did not produce any evident change in the eye (Figure 7J‐L), yet co‐expression of Lic and Hep resulted in eyes with drastically reduced size (Figure 7M), while co‐expression of Lic and Bsk failed to produce this phenotype (Figure 7N). Interestingly, loss of *lic *could impede ectopic dTAK1‐ but not Wnd‐triggered small eye phenotype (Figure S10), suggesting Lic mediates dTAK1‐induced cell death. Based on the above evidences, we conclude that Lic most probably acts in parallel with Hep to promote JNK‐mediated cell death.

As Lic has previously been reported as a MAPKK for the p38 kinase, we examined whether Lic activates JNK signalling through p38, or via a mechanism that is independent of p38. The *Drosophila *genome encodes three p38 family members, designated as p38a, p38b and p38c,^43^ with p38b being proposed to play a central role in *Drosophila* p38 signalling.^44^ We found that *ptc*>Lic‐induced loss‐of‐ACV phenotype was not affected by knocking‐down *p38a*, *p38b* or *p38c* (Figure 7F,I and Figure S9d‐g), suggesting Lic regulates JNK‐mediated cell death in a p38‐independent manner.

### 3.9 Lic modulates physiological JNK activity

**Figure 8:**
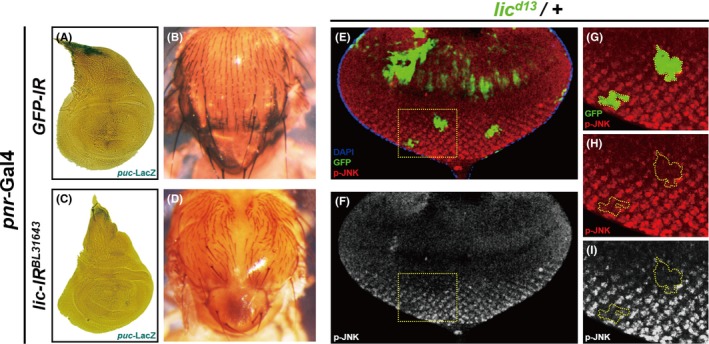
Lic is required for physiological JNK activation. Light micrographs of *Drosophila* wing discs (A, C) and adult thoraxes (B, D) are shown. The endogenous *puc* expression pattern in the notum region of wing disc (A) is impeded by knock‐down *lic* (C). Compared with the control thorax (B), loss of *lic* produces a thorax cleft phenotype (D). Confocal images of mosaic eye imaginal discs (E‐I) are shown. *lic* mutant clones were generated with the MARCM system and labelled by GFP expression (green). Localized p‐JNK signal is detected in the posterior of eye disc and is lost in *lic* mutant clones (G‐I). G‐I are high magnification views of the boxed area in E. Genotypes: (A) *GFP‐RNAi*/*+*; *pnr*‐Gal4/*puc*‐LacZ (B) *GFP‐RNAi*/*+*; *pnr*‐Gal4/*+* (C) *pnr*‐Gal4, *UAS‐lic‐RNAi^BL31643^*/*puc*‐LacZ (D) *pnr*‐Gal4/*UAS‐lic‐RNAi^BL31643^* (E‐I) *lic^d13^*, *FRT19A*/*tub*‐Gal80, *hs‐Flp*, FRT19A; *act*‐Gal4, *UAS*‐GFP/+

Endogenous JNK signalling is required for the thorax closure process in normal development, while impaired JNK activity results in a thorax cleft phenotype in the adults.^10^, ^45^ Downregulation of *lic *under the *pnr* promoter resulted in reduced *puc *expression in the dorsal tip of wing disc (Figure 8A,C) and produced a cleft phenotype in the adult thorax (Figure 8B,D), suggesting Lic modulates the physiological functions of JNK signalling. Endogenous JNK activity could be detected by p‐JNK staining in the eye disc posterior to the morphogenetic furrow (Figure 8E,F). We generated homozygous *lic* mutant clones in the eye disc using the mosaic analysis with a repressible cell marker (MARCM) system. Intriguingly, p‐JNK staining is dramatically reduced in homozygous *lic^d13^* mutant clones (Figure 8E‐I), suggesting that Lic is essential for endogenous JNK activation.

## 4 DISCUSSIONS

*lic* encodes the *Drosophila* ortholog of MKK3, which has been previously reported as the MAPK kinase modulating p38 signalling in cell growth, stress response, innate immunity and asymmetric egg development in oogenesis.^46^, ^47^, ^48^, ^49^ Here, we provide compelling genetic evidences to conclude that *lic *encodes an essential component of the Egr‐JNK pathway involved in cell death. We showed that Lic is necessary for ectopic Egr‐triggered JNK‐mediated cell death in eye and wing development. In addition, Lic is required for loss‐of‐cell polarity‐induced JNK‐dependent cell death. Moreover, ectopic Lic is sufficient to trigger JNK‐dependent cell death in multiple tissues. Furthermore, Lic is necessary and sufficient for Egr‐induced JNK activation. Finally, Lic acts in parallel with Hep to promote JNK phosphorylation. Although Lic was previously reported as a MAPKK for the p38 kinase, our data suggest that Lic activates JNK in cell death via a mechanism that is independent of p38. Firstly, loss of *lic*, but not *p38*, suppressed Egr‐triggered JNK‐mediated cell death (Figure 1D,E,S and data not shown). Secondly, ectopic Lic‐induced JNK activation and cell death were not affected by loss of p38 (Figure 7F,I and data not shown). Thirdly, Lic acts in parallel with Hep, as depletion of *hep* failed to impede ectopic Lic‐triggered cell death, and *vice versa* (Figure 7E,H), whereas co‐expression of Lic and Hep exhibited synergistic effect in promoting cell death (Figure 7M).

The p38 mitogen‐activated protein kinase (MAPK) pathway is activated in response to a variety of environmental stresses.^50^ Although p38 signalling has been previously implicated in apoptosis, contradictory results suggest p38 could function as a positive or negative regulator.^51^, ^52^, ^53^ JNK signalling plays a pro‐apoptotic role in both mammalian and *Drosophila *systems.^54^ Our study shows that ectopic expression of Lic promotes JNK phosphorylation and *puc* transcription, both are read‐out of JNK signalling activation. Lic‐induced apoptosis, which is independent of p38 but JNK‐dependent, reminds us to pay more attention to the role of Lic in cell death and other JNK‐related physiological process such as tumour development. Finally, it remains intriguing whether MKK3 participates in JNK signalling in mammals.

## CONFLICT OF INTEREST

The authors declare no conflict of interest.
