# Supplementary material for: Lic regulates JNK‐mediated cell death in Drosophila
Source: Cell Prolif. 2019 Mar 7;52(3):e12593. doi: 10.1111/cpr.12593 (PMC6536442; doi:10.1111/cpr.12593)
Supplement: Supplementary file 1 [file CPR-52-e12593-s001.docx]

**Supplementary Figures**


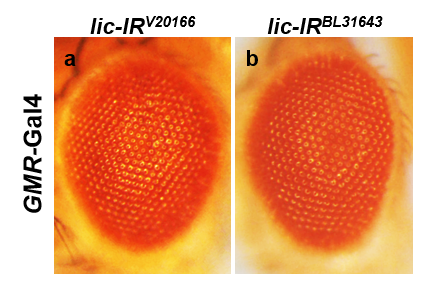
**Sun et al., Fig S1**

**Figure S1. Depletion of *lic* does not affect eye size**

Light micrographs of *Drosophila* adult eyes are shown. Expression of two independent *UAS-lic-RNAi* lines driven by *GMR*-Gal4 produced no obvious eye phenotype. Genotypes: (a) *GMR*-Gal4/*UAS-lic-RNAi^V20166^* (b) *GMR*-Gal4/+; *UAS-lic RNAi^BL31643^/+*.

**Sun et al., Fig S2**

**
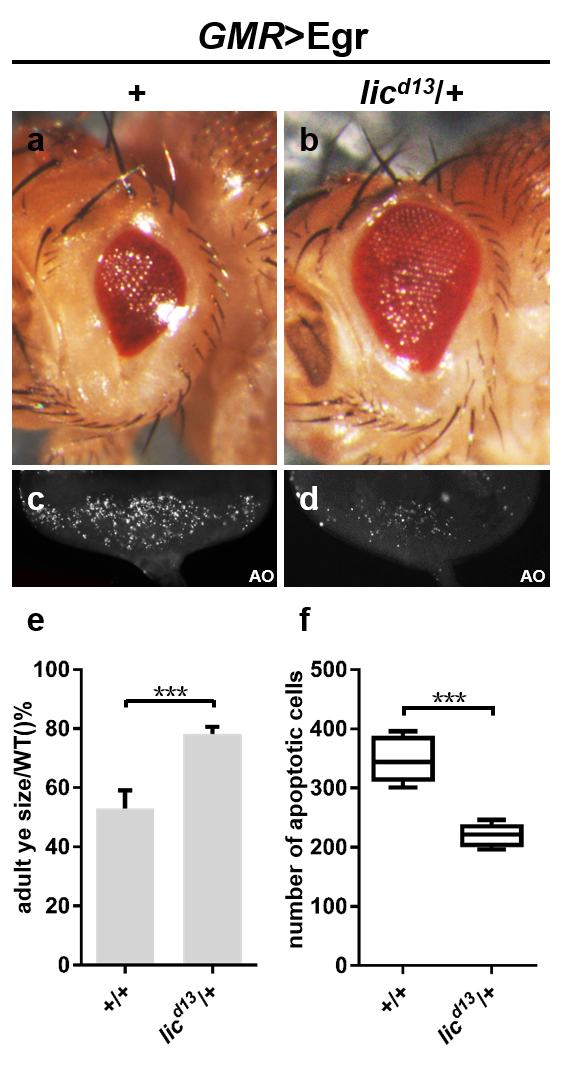
**

**Figure S2. Heterozygous *lic* mutation suppresses ectopic Egr-induced cell death in eye development**

Light micrographs of *Drosophila* adult eyes (a, b) and fluorescent micrographs of *Drosophila* third instar eye discs (c, d) are shown. *GMR*>Egr-induced small eye phenotype in adults (a) and massive cell death in 3rd instar larval eye discs (c) are suppressed in heterozygous *lic* mutants (b, d). (e) Quantification of adult eye sizes, which have been normalized to the *GMR*-Gal4 control, are shown, *n*≥10. (f) Statistic analysis of AO-positive cell numbers per disc, *n*≥10. Student-t test was used to calculate statistical significance, mean + SEM, ***, *P*<0.001. Genotypes: (a, c) *UAS*-Egr/+; *GMR*-Gal4/+ (b, d) *lic^d13^*/+; *UAS*-Egr/+; *GMR*-Gal4/+.

**Sun et al., Fig S3**


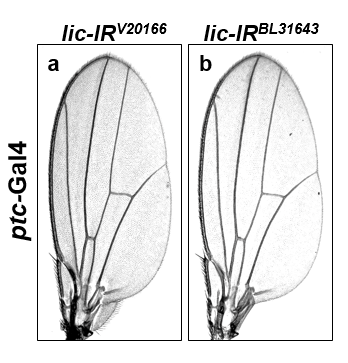


**Figure S3. Depletion of *lic* does not affect wing development**

Light micrographs of adult wings are shown. Expression of two independent *UAS*-*lic-RNAi* lines driven by *ptc*-Gal4 does not produce obvious wing phenotype. Genotypes: (a) *ptc*-Gal4/*UAS-lic-RNAi^V20166^* (b) *ptc*-Gal4/+; *UAS-lic-RNAi^BL31643^/+*.

**Sun et al., Fig S4**


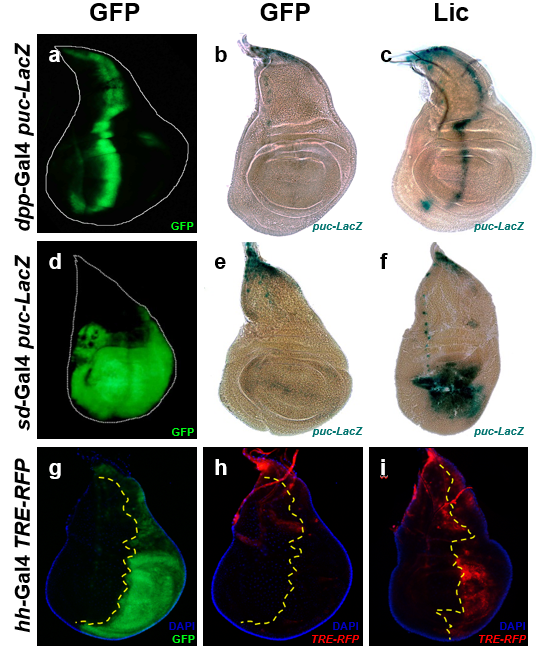


**Figure S4. Overexpression of Lic induces JNK pathway activation**

Fluorescent (a, d, g-i) and light (b, c, e, f) micrographs of *Drosophila* third instar wing discs are shown. GFP depicted the region driven by *dpp-, sd-* or *hh*-Gal4 (a, d, g). Ectopic expression of Lic (c, f, i), but not GFP (b, e, h) activated *puc*-LacZ (b, c, e, f) or *TRE*-RFP (h, i) expression in the corresponding regions. The *TRE*-RFP reporter has the TRE sequence placed in front of the RFP coding sequence, so that the RFP signal served as a readout of JNK pathway activation. Genotypes: (a) *UAS*-GFP/+; *dpp*-Gal4/*+* (b) *UAS*-GFP/+; *dpp*-Gal4/*puc*-LacZ (c) *dpp*-Gal4, *UAS*-Lic/*puc*-LacZ (d) *sd*-Gal4; *UAS*-GFP/+ (e) *sd*-Gal4; *puc*-LacZ/*UAS*-GFP (f) *sd*-Gal4; *puc*-LacZ/*UAS*-Lic (g) *hh*-Gal4, *UAS*-GFP/+ (h) *TRE*-RFP/+; *hh*-Gal4, *UAS*-GFP/+ (i) *TRE*-RFP/+; *hh*-Gal4, *UAS*-GFP /*UAS*-Lic.

**Sun et al., Fig S5**


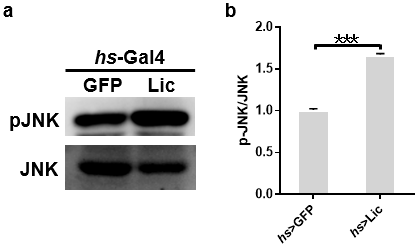


**Figure S5. Overexpression of Lic promotes JNK phosphorylation**

Ectopic Lic up-regulates the phosphorylation level of JNK as shown by Western blot (a). The phosphorylation levels of JNK are normalized to the total JNK levels (b). Statistical significance is determined with Student’s t test, ***, *P*< 0.001.

**Sun et al., Fig S6**


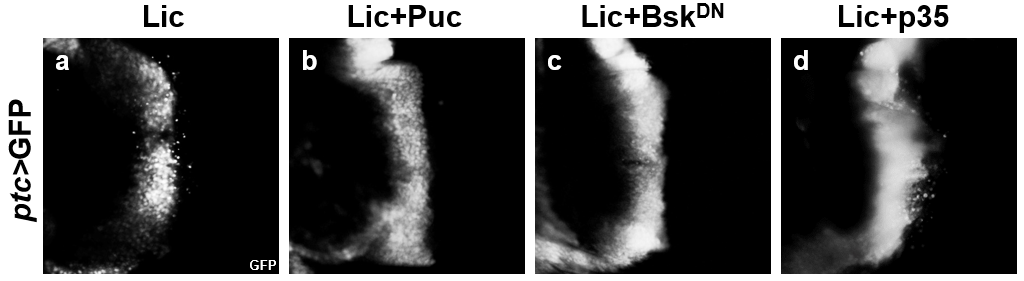


**Figure S6. Lic induces JNK-dependent cell death and migration**

Fluorescent micrographs of *Drosophila* third instar wing discs are shown. The *ptc*>GFP stripe was narrowed and exhibited cell migration upon Lic expression (a), both of which were strongly impeded by expression of Puc (b) or Bsk^DN^ (c). Expression of p35 only rescued the narrowed GFP stripe but not cell migration triggered by Lic (d). Genotypes: (a) *ptc*>GFP/+; *UAS*-Lic/+ (b) *ptc*>GFP/+; *UAS*-Lic/*UAS*-Puc (c) *ptc*>GFP/+; *UAS*-Lic/*UAS*-Bsk^DN^ (d) *ptc*>GFP/+; *UAS*-Lic/*UAS*-p35.

**Sun et al., Fig S7**


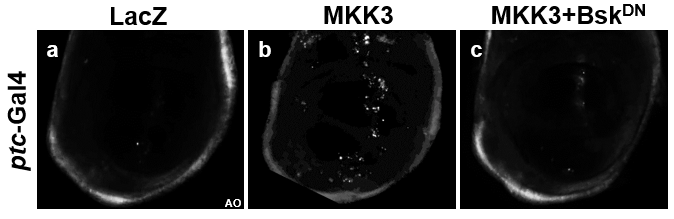


**Figure S7. MKK3 induces JNK-dependent cell death in the wing disc**

Fluorescent micrographs of third instar wing discs are shown (a-c). Compared with the control (a), expression of MKK3 induced extensive cell death (b) that was suppressed by Bsk^DN^. Genotypes: (a) *ptc*-Gal4/+; *UAS*-LacZ/+ (b) *ptc*-Gal4/+; *UAS*-MKK3/+ (c) *ptc*-Gal4/+; *UAS*-MKK3/*UAS*-Bsk^DN^.


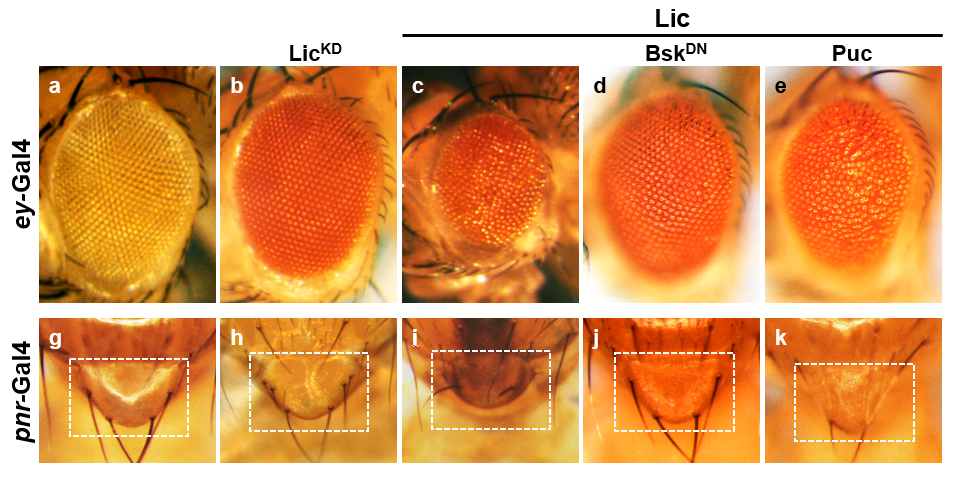
**Sun et al., Fig S8**


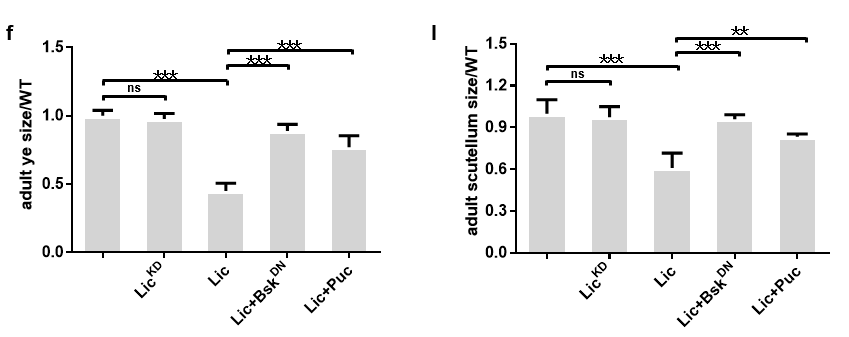


**Figure S8. Lic induces JNK-dependent cell death in a non-tissue specific manner**

Light micrographs of *Drosophila* adult eyes (a-e) and thoraxes (g-k) are shown. The box depicts the scutellum. Compared with controls (a, g), expressing Lic in the eye driven by *ey*-Gal4 or in the dorsal midline by *pnr*-Gal4 produced a small eye or scutellum phenotype (c, i), both of which were restored by expressing Bsk^DN^ (d, j) or Puc (e, k). Expression of Lic^KD^ failed to trigger any significant phenotype (b, h). Statistical analysis of eye (f) or scutellum (l) size, which were normalized to the controls, was shown. One-way ANOVA test was used to calculate statistical significance, n≥9, mean + s.d., ns, *P*>0.05; **, *P*<0.01; ***, *P*<0.001. Genotypes:

(a) *ey-*Gal4/+ (b) *ey-*Gal4/+; *UAS*-Lic^KD^/+ (c) *ey-*Gal4/+; *UAS*-Lic/+ (d) *ey-*Gal4/+; *UAS*-Bsk^DN^/*UAS*-Lic (e) *ey-*Gal4/+; *UAS*-Puc/*UAS*-Lic (g) *pnr*-Gal4/+ (h) *pnr*-Gal4/*UAS*-Lic^KD^ (i) *pnr*-Gal4, *UAS*-Lic/+ (j) *pnr*-Gal4, *UAS*-Lic/*UAS*-Bsk^DN^ (k) *pnr*-Gal4, *UAS*-Lic/*UAS*-Puc.

**Sun et al., Fig S9**

**
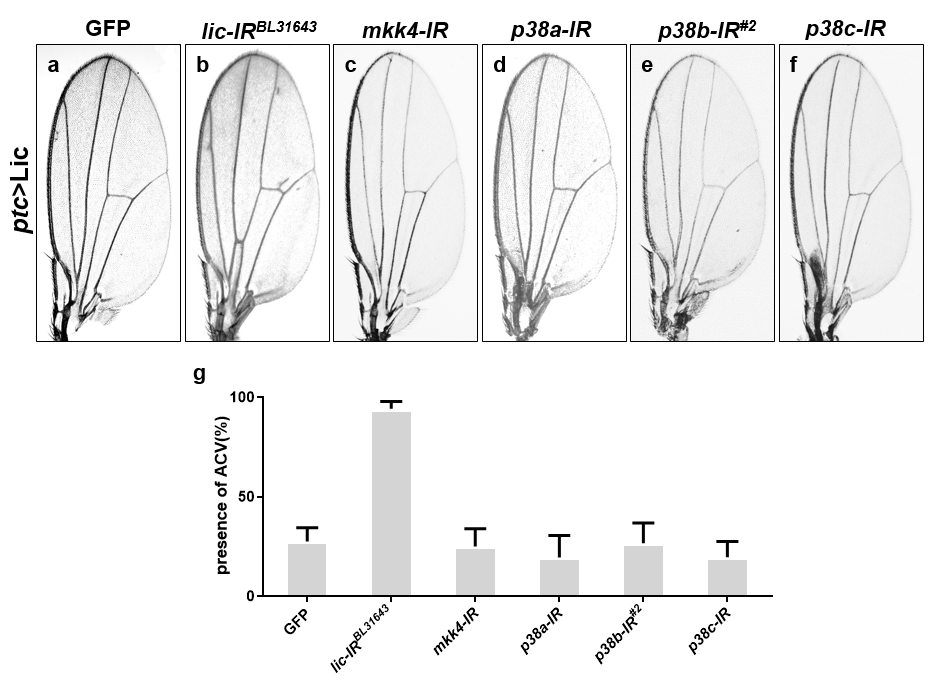
**

**Figure S9. Lic provokes p38-independent cell death**

Light micrographs of *Drosophila* adult wings are shown (a-f). Ectopic Lic-triggered ACV loss was blocked by expressing a *lic* RNAi (b), but remained unaffected by expressing GFP (a), or RNAi of *mkk4* (c), *p38a* (d), *p38b* (e) or *p38c* (f). The presence of ACV in adult wings were quantified (g). Genotypes:

(a) *ptc*>GFP/+; *UAS*-Lic/+ (b) *ptc*-Gal4/+; *UAS*-Lic/*UAS-lic-RNAi^BL31643^* (c) *ptc*-Gal4/*UAS-mkk4-RNAi*; *UAS*-Lic/+ (d) *ptc*-Gal4/+; *UAS*-Lic/*UAS-p38a-RNAi* (e) *ptc*-Gal4/+; *UAS*-Lic/*UAS-p38b-RNAi^#2^* (f) *ptc*-Gal4/+; *UAS*-Lic/*UAS-p38c-RNAi*.

**Sun et al., Fig S10**


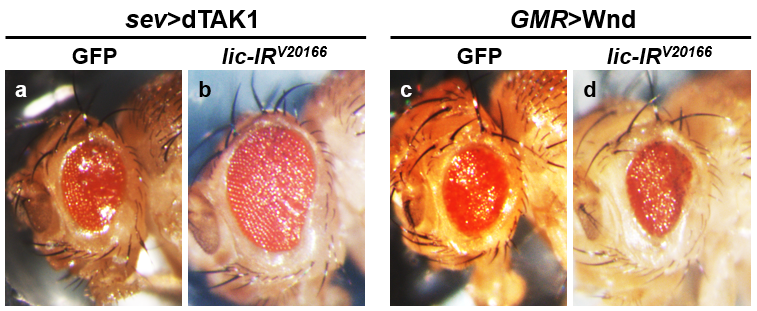


**Figure S10. Lic mediates dTAK1-induced cell death**

Light micrographs of *Drosophila* adult eyes are shown. Expressing dTAK1 by *sev*-Gal4 (*sev*>dTAK1) produced a small and rough eye phenotype (a), which was suppressed by *lic* depletion (b). Ectopic Wnd-induced small-eye phenotype (c) is not suppressed by loss of *lic* (d). Genotypes: (a) *sev>*dTAK1/+; *UAS*-GFP/+ (b) *sev>*dTAK1/*UAS-lic-RNAi^V20166^* (c) *GMR*>Wnd/*UAS-*GFP (d) *UAS-lic-RNAi^V20166^*/+; *GMR*>Wnd/+.
